# Acute Kidney Injury and Organ Dysfunction: What Is the Role of Uremic Toxins?

**DOI:** 10.3390/toxins13080551

**Published:** 2021-08-09

**Authors:** Jesús Iván Lara-Prado, Fabiola Pazos-Pérez, Carlos Enrique Méndez-Landa, Dulce Paola Grajales-García, José Alfredo Feria-Ramírez, Juan José Salazar-González, Mario Cruz-Romero, Alejandro Treviño-Becerra

**Affiliations:** 1 Department of Nephrology, General Hospital No. 27, Mexican Social Security Institute, Mexico City 06900, Mexico; dr.ivanlarap@gmail.com (J.I.L.-P.); dgrajalesgarcia@gmail.com (D.P.G.-G.); 2 Department of Nephrology, Specialties Hospital, National Medical Center “21st Century”, Mexican Social Security Institute, Mexico City 06720, Mexico; drcruznefro@gmail.com; 3 Department of Nephrology, General Hospital No. 48, Mexican Social Security Institute, Mexico City 02750, Mexico; ashkeloningvar@gmail.com; 4 Department of Nephrology, General Hospital No. 29, Mexican Social Security Institute, Mexico City 07910, Mexico; dr.alfredferia@gmail.com; 5 Department of Nephrology, Regional Hospital No. 1, Mexican Social Security Institute, Mexico City 03100, Mexico; juan.salazargo@imss.gob.mx; 6 National Academy of Medicine, Mexico City 06720, Mexico; atreve16@yahoo.com.mx

**Keywords:** uremic toxins, acute kidney injury, organ failure, cardiovascular disease, lung injury

## Abstract

Acute kidney injury (AKI), defined as an abrupt increase in serum creatinine, a reduced urinary output, or both, is experiencing considerable evolution in terms of our understanding of the pathophysiological mechanisms and its impact on other organs. Oxidative stress and reactive oxygen species (ROS) are main contributors to organ dysfunction in AKI, but they are not alone. The precise mechanisms behind multi-organ dysfunction are not yet fully accounted for. The building up of uremic toxins specific to AKI might be a plausible explanation for these disturbances. However, controversies have arisen around their effects in organs other than the kidney, because animal models usually depict AKI as a kidney-specific injury. Meanwhile, humans present AKI frequently in association with multi-organ failure (MOF). Until now, medium-molecular-weight molecules, such as inflammatory cytokines, have been proven to play a role in endothelial and epithelial injury, leading to increased permeability and capillary leakage, mainly in pulmonary and intestinal tissues.

## 1. Introduction

AKI is defined as a rapid increase in serum creatinine, a reduced urinary output, or both. It occurs in nearly 15% of in-hospital patients, but its occurrence rises significantly (up to 50%) in critical settings, such as the intensive care unit (ICU) [1]. Despite recent progress in understanding its pathophysiology and renal replacement therapies, mortality related to AKI remains considerably high, which may be due to non-renal effects of AKI [2]. 

AKI definition has evolved, from RIFLE criteria in 2002, later revisited by the Acute Kidney Injury Network (AKIN), until the most recent consensus by Kidney Disease Improving Global Outcomes (KDIGO). Currently, it is defined as an increase in serum creatinine (SCr) ≥ 1.5 times the baseline, an increase in SCr ≥ 0.3 mg/dL within any 48 h or a urinary output less than 0.5 mL/kg/hr for at least 6 h. The duration must be ≤7 days. Acute kidney disease (AKD) refers to subacute damage or loss of kidney function for a duration between 7 and 90 days after an AKI-initiating event. Chronic kidney disease (CKD) appears when damage remains after 90 days [3].

During an event of AKI, the reduced excretory abilities of the kidneys may lead to the accumulation of several solutes. Those which exert some biological activities are termed uremic toxins. A diverse range of uremic toxins has been identified, some of which have an impact on cardiovascular mortality in patients with chronic kidney disease (CKD) in pre-dialysis care. However, their effects in AKI remain controversial [4] and could represent a likely explanation for organ failure in AKI [5].

AKI may occur prior to, or as a consequence of, multiple organ failure (MOF). The pathophysiology of AKI encompasses a broad spectrum of clinical associations, each with its unique mechanism driving kidney damage. A significant proportion of patients in the ICU present with multiple comorbidities (i.e., shock, sepsis, trauma, and surgery) which place them at higher risk of MOF. An imbalance between oxygen supply and demand may cause hypoxia, initiating a series of cellular derangements that include lactic acid production, microcirculatory and mitochondrial dysfunction, all of which may drive organ failure in critical patients [6].

Furthermore, a highly frequent component of AKI is a reduction in renal blood flow, which, when restored, may trigger ischemia-reperfusion injury (IRI) [1,6]. IRI in the kidney has been shown to increase levels of ROS, altering mitochondrial pathways, leading to ATP depletion, cytosolic calcium release, caspase activation, and oxidative-damage-targeting lipids, DNA and proteins, ultimately driving cell death. During IRI, antioxidant mechanisms are downregulated (such as catalase, superoxide dismutase and glutathione peroxidase) [7]. Cytokines released during tissue damage include IL-6, TNFα, JAK/STAT, and enhanced expression of ICM-1 and P-selectin promotes neutrophil and macrophage migration and activation. Additionally, there is an upregulation of the renin-angiotensin system including hemodynamic and apoptosis-inducing effects, as well as complement-mediated cell damage [8].

An initial insult (hypoxia, IRI, hypovolemia, etc.) may cause organ failure, with AKI being one of the consequences; however, this does not exclude a potential role for individual toxins aggravating distant organ failure. These conditions may increase production of toxins, which accumulate in the setting of decreased urinary clearance, thus making it difficult to conduct studies regarding the effects of any given uremic toxin in a specific organ [9]. Animal models of AKI have provided some insight into the possible effects of certain toxins on other organs. Studies have shown an interaction with the blood–brain barrier, gut microbiota, lung epithelia and the cardiovascular system. 

Clinical conditions associated with IRI include kidney transplantation, sepsis, shock, surgery and exposure to nephrotoxins. Some uremic toxins have been proven to be associated with negative outcomes in critically ill patients. If patients with AKI could benefit from the removal of certain toxins with renal replacement therapy (RRT) is still uncertain. Given the high burden of complications in these settings, research is needed to provide possible therapeutic targets and guide clinicians in order to improve patient outcomes. 

## 2. AKI: Beyond the Kidney

Physiological derangements occur throughout the continuum of AKI, AKD and CKD; they occur during AKI at a naturally accelerated pace. Toxin accumulation is not uniform in these scenarios, as shown in several studies that have found lower levels of certain toxins in AKI than in CKD. The systemic inflammatory response syndrome drives sepsis-associated AKI, in which endothelial and epithelial injury result in increased permeability, capillary leak and transfer of substances through the intestinal membrane to several organs [10]. Additionally, it is possible that the increase in some toxins may be due to concomitant hepatic or gastrointestinal dysfunction [11]. The significance of studying these toxins in AKI is to determine which may contribute to non-kidney organ dysfunction, and which may be associated with endothelial or tubular injury, possibly having an effect on renal recovery [12]. Uremic toxins are classified by EuTox (European Uremic Toxin Work Group), as shown in Table 1 [13].

Presently, metabolic serum analysis using liquid chromatography and mass spectrophotometry have demonstrated that metabolomics might be useful to identify uremic toxins [14]. Given that AKI is a high-priority medical condition and is a frequent complication in critical settings, few studies have focused on the measurement of specific uremic toxins, which contributes to a lack of research in this particular field.

Defining the characteristics of distinct uremic toxins is challenging, given that substances within a category do not possess similar biological effects. Furthermore, there are no set values above which they present adverse effects [13,14]. Some effects of uremic toxins may target mitochondria, thus altering redox homeostasis, disturbing cellular mechanisms in multiple organs. Additionally, it has been proposed that up to 30% of uremic toxins may be produced or cleared by mitochondria [15,16].

Animal models of AKI explore the specific pathology of the kidney in particular. On the other hand, human AKI may be driven in association with hypoxia, which in turn may trigger MOF [17,18], making it difficult to discern the complex relationships between one particular toxin and its possible effects, as well as the contribution of individual organs in MOF Clinical nephrologists guide management by traditional indications for RRT, and the levels of the uremic toxins described in this review are not measured routinely [18,19].

**Figure 1:**
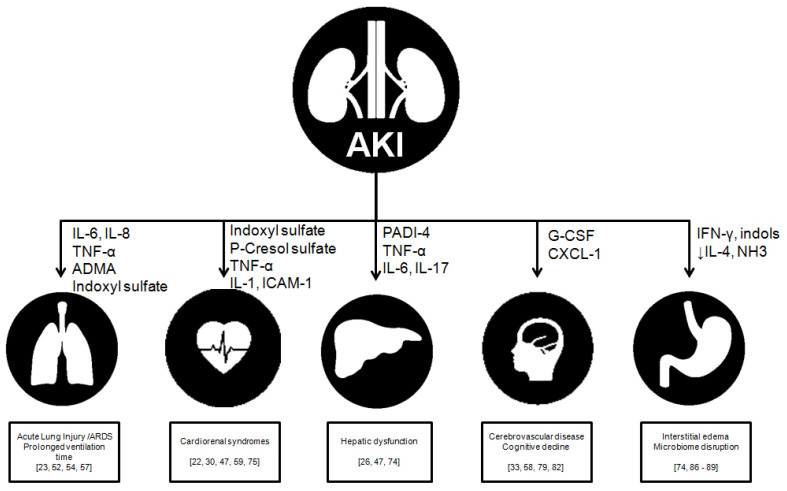
Interactions, clinical outcomes and responsible uremic toxin. ARDS (acute respiratory distress syndrome), TNF-α (tumor necrosis factor α), ICAM-1 (circulating intercellular adhesion molecule-1), IL-4 (interleukine-4), IL-6 (interlekin-6), IL-6 (interleukin-6), IL-8 (interleukin-8), IL-17 (interleukin-17), ADMA (asymmetric dimethyl arginine), G-CSF (Granulocyte colony-stimulating factor), PADI 4 (protein-arginine deiminase type-4), and IFN-γ (Interferon-gamma).

The term crosstalk put forward by Lee et al. refers to AKI originating dysfunction of ‘distant’ organs, i.e., lungs, heart, brain, liver and intestine. This occurs through deranged organ communication (Figure 1). A balance between immunological cytokines and cellular components is important because they mediate organ crosstalk in AKI [17].

## 3. Small Water-Soluble Substances

### 3.1. Asymmetric Dimethyl-Arginine (ADMA)

ADMA originates during methionin metabolism and is the main endogenous regulator (through competitive inhibition) of nitric oxide synthase (NOS), an enzyme converting L-arginine to nitric oxide and L-citrulline. AKI alters the metabolic pathways of arginine and methylarginine, resulting in a reduced availability for nitric oxide in the kidney and circulation. The excess ADMA is inactivated enzymatically by dimethylarginine dimethylaminohydrolase (DDAH), widely expressed in the kidney [20,21].

In a study by Sun et al., comparing serum samples of 17 patients with AKI, a 91-fold increase in ADMA levels was found in patients with AKI compared to age-matched subjects with preserved kidney function [14]. Increased levels of ADMA may lead to endothelial dysfunction and an increased permeability via mitogen activated protein kinase p38/heat-shock protein 27 (MAPK/HSP 27); it is, thus, implicated in the progression to acute kidney disease (AKD) and chronic kidney disease [22].

Animal models have proven that elevated HSP27 correlates to acute lung injury accompanying AKI. Ma and Liu have developed a model for ischemic AKI using Winstar rats undergoing bilateral continuous renal vascular occlusion. They measured plasmatic ADMA using immune adsorption, and MAPK/HSP27 through Western blot in rats with induced AKI and a control group. Levels of ADMA and expression of p38 and phosphorylated HSP27 were significantly elevated in the AKI group, compared to controls. Additionally, rats with induced AKI developed epithelial edema, proliferation of the alveolar walls and capillary congestion, concluding that HSP27 elevations are implicated in AKI development and possibly correlate with acute lung injury [23].

Besides, a role has been proposed for liver-regulated ADMA levels. Carnegie et al. envisioned this possibility when finding diminished kidney ADMA expression in patients with chronic hepatitis [24]. Liver injury in the critical ill patient is independently correlated with ADMA concentrations, proving that this toxin is not limited to chronic diseases, and may be relevant in multiorgan failure in critical settings [25].

Through a limited renal or hepatic clearance, severe inflammation leads to an increased proteolysis, diminished liver and kidney DDAH expression, and consequently reduced urinary ADMA, leading to its accumulative effects through NOS inhibition and reduced nitric oxide. This leads to organ injury via endothelial dysfunction, reduced blood flow and increased inflammatory response [26].

Recently, levels of soluble uremic toxins and urinary biomarkers for AKI were studied in runners without kidney disease [27]. Traditional markers (urea and creatinine) and symmetric DMA were found to be elevated, as opposed to ADMA. Urinary biomarkers included urinary NGAL, Cystatin C and KIM-1 and may help distinguish exercise-induced AKI from a physiological response to strenuous exertion.

### 3.2. Guanidine Compounds

Arginine is an amino acid involved in the metabolism of nitric oxide, urea, polyamines, agmatine and other related compounds (guanidinoacetic acid, creatine, creatinine), as well as methylated, hydrolyzed and oxidized substances. Some organs participating in this metabolic pathway are the small intestine, kidneys, liver and skeletal muscle. In the small intestine, glutamine is converted to citrulline, which is transported to the kidney and metabolized to arginine in the proximal convoluted tubule. Thus, arginine provides for guanidinoacetic acid, creatine and guanidinosuccinic acid, which are in turn delivered to specific organs (brain, heart, and skeletal muscle) to become substrates for ATP synthesis [28]. These metabolic communications between kidneys, intestine and skeletal muscle suggest that an injury to any organ may dramatically impair the metabolic pathways of arginine [29].

Guanidine compounds induce an inflammatory response by activating leukocytes and stimulating their proliferation in vitro; on the contrary, these substances inhibit the functions of previously activated leukocytes [30,31].

Levillain et al. [32] performed experiments on rats undergoing subtotal nephrectomy (72% of the kidney tissue), and measured plasma levels of guanidine compounds. During the first 48 h, an increased plasma citrullin and diminished Arginine synthase activity was observed, due to the impairment of kidney ability to produce arginine and guanidinoacetic acid. Later, in the recovery phase, guanidine compounds were elevated because of a higher citrullin availability. Therefore, these substances behave differently in AKI and CKD; during the progression of AKI to CKD, a myriad of physiologic and metabolic mechanisms lead to an increase in glomerular filtration rate (GFR), new expression of enzyme precursors and accumulation of guanidine compounds in affected tissues [32].

In humans, guanidine compounds may accumulate in cerebrospinal fluid of uremic patients. In turn, these substances may predispose to seizures by antagonizing GABA and activating NMDA receptors. They may even play a role in blood pressure regulation on the rostral ventrolateral medulla [33].

### 3.3. Uric Acid

In a simple fashion, the increased uric acid levels in AKI are a byproduct of diminished GFR, leading to reduced excretion, and thus might be a marker for the severity of the injury. Nonetheless, this increase in uric acid may confer detrimental effects [34].

Uric acid mediated vasoconstriction has been shown in rat models of hyperuricemia, and is marked by an increase in afferent arteriole resistance and associated decline in GFR. This mechanism appears independent from nitric oxide, since vasoconstriction is reversed with L-arginine [35]; it has also been shown that uric acid inhibits the release of nitric oxide from endothelial cells and treatment with allopurinol increases plasma nitrite levels [36].

This has indirectly been proven in humans, when measuring the brachial artery reactivity (a surrogate for altered endothelial NO release). Uric acid increases the release of monocyte chemoattractant protein-1 (MCP-1) in smooth vascular muscle, and the synthesis of C-reactive protein in smooth and vascular human tissue [37].

A reduction in uric acid improves endothelial function in patients with asymptomatic hyperuricemia [38], congestive heart failure [39], diabetes [40] and hypercholesterolemia [41].

In summary, uric acid effects include renal vasoconstriction (via NOS1 inhibition, a reduced release of NO from endothelium, and activation of renin-angiotensin system), antiangiogenic properties (reduced proliferation and migration, increased apoptosis of endothelial cells), pro-inflammatory effects (stimulation of MCP-1 and CRP, activation of NF-KB and p38-MAPK) and oxidative stress (elevated ROS and peroxinitrile) [38,39,40,41,42]. Still, uric acid is also considered the most abundant antioxidant in human plasma. Specifically, uric acid may bind to superoxide anion and peroxinitrile, helping maintain levels of extracellular superoxide dismutase by preventing its downregulation. Yet, this beneficial effect is overrun in states of severe hyperuricemia such as AKI [34,43].

Loop diuretics, widely prescribed in AKI management, promote uric acid reabsorption in the proximal tubule, via direct effects of the drugs on epithelial transporters and indirectly by reducing kidney perfusion, which in turn stimulates proximal tubule sodium and urate transport [44].

Given this controversy of uric acid in AKI, Srivastava et al. [45] aimed to measure serum uric acid in 458 patients admitted to the ICU, and 250 patients under RRT from a cohort of patients enlisted in the Acute Renal Failure Trial Network (ATN). However, they fail to find a statistically significant association between serum uric acid levels and 60-day mortality, in unadjusted model or after multivariable analysis controlling for demographics, disease severity and kidney-specific (OR adjusted by doubling of uric acid levels, 1.15 (95% CI: 0.71–1.86).

## 4. Protein-Bound Uremic Toxins

A growing body of evidence supports the role of a disturbance in the intestinal barrier and gut-derived toxins (phenol, indole), as well as toxins ingested through dietary intake that may have direct cardiovascular effects [46].

### 4.1. Indoxyl Sulfate (IS)

IS is a low-molecular-weight uremic toxin product of tryptophan metabolism that has kidney clearance by proximal tubule secretion via organic anion transporters (OAT1 and 3), thus accumulating in states of AKI and CKD [47,48]. Values higher than 2.74 μg/mL have been clinically associated with 90-day mortality after hospital-acquired AKI [49,50].

Proven effects of IS include an increase in inflammatory markers, oxidative stress markers, collagen synthesis and inhibition of endothelial proliferation, leading to tubular toxicity, interstitial fibrosis, glomerular sclerosis and kidney function decline [47]. These effects are mediated via cytokine expression (i.e., cell adhesion molecule 1, transforming growth factor beta1 and plasminogen activator inhibitor-1 [51].

A major contributor to mortality in AKI is linked to acute lung injury, which is a frequent complication in this setting, and these disturbances are immensely related to mortality. Aquaporin-5 is a channel responsible for water diffusion through the apical membrane of alveolar type I cells. The expression of Na+/K+ ATPase is regulated by factors related to ischemia, as well as inflammatory markers, more so than uremic toxins. It is possible that uremic toxins alter the expression of Na/K+ ATPase proteins [52].

Increased IS levels were reported previously in rat model (nephrectomy 5/6) as well as cisplatin-induced AKI [53]. Yabuuchi et al. [54] studied the role of IS in AQP-5 lung expression in rats undergoing bilateral nephrectomy. Using Western Blot, they proved a significant reduction in AQP-5 expression, which was reversed by oral administration of AST-120 (spherical particles containing activated charcoal) [55,56]. Comparatively, no significant change was evident in Na+/K+ ATPase expression. These findings were supported by immunohistochemistry analysis. The building up of IS may alter AQP-5 expression in the lung via activation of p38 MAPK and JNK, which in turn lead to thickening of interstitial lung tissue [57].

Iwata et al. [58] measured IS levels in rats with cisplatin-induced AKI at 24 and 48 h intervals. Likewise, they found elevated IS levels correlate with elevated serum creatinine and urea nitrogen. Surprisingly, the levels of all three toxins decreased after treatment with AST-120. Plus, they found elevated IS levels in brain tissue, suggesting a link between the central nervous system and the kidney [58].

With regard to the cardiovascular system, IS presents pro-fibrotic and pro-hypertrophy effects in heart muscle cells in patients with CKD [59]. A study in 26 male patients with AKI explored the endothelial effects of IS [60]. This study found a reduced number of circulating endothelial progenitor cells, which upon exposure to IS, have reduced expression of nitric oxide synthase and cell adhesion molecules, which impairs their ability to migrate and proliferate. Statins may have a therapeutic role, since atorvastatin exposure reversed the changes on progenitor cells.

A study of 194 adult patients with AKI related to sepsis found that changes in levels of IS and p-cresyl may differ [61]. Patients with persistent AKI have a moderate rise in p-cresyl, but no change in IS levels. Patients whose AKI resolved were shown to have decreased levels of all solutes.

### 4.2. Homocysteine

Up to 3.5 times higher levels of homocysteine have been found in individuals with AKI compared to healthy subjects [13]. The proposed mechanisms for this finding include reduced kidney clearance, uremia-mediated inhibition of key enzymes in the methionin-homocystein metabolism (i.e., homocystein methyltransferase) as well as relative or absolute deficiencies of folate, vitamin B6 or vitamin B12 [62].

So far, data have been collected from animal models exploring the effect of hyperhomocysteinemia in AKI. In these models of cisplatin-induced AKI, homocysteine levels above 30 μM were achieved, finding damage to tubular cells by inducing apoptosis and inhibiting proliferation. However, it is not clear whether the damage can be attributed to homocysteine or cisplatin, which is why the role of this toxin in AKI is not conclusive [63].

## 5. Medium Molecular Weight Molecules

### 5.1. Proinflammatory Cytokines

Inflammation is intimately close to AKI, in a complex network of interactions between parenchymal kidney tissue, local immune cells (macrophages and dendritic cells) and migrating cells (monocytes, lymphocytes and circulating neutrophils) as well as specialized cell receptors (Toll-like or Nod-like) [64]. Cytokines are defined as a heterogeneous group of cell signaling proteins weighing between 5 and 20 kDa, and controversy has risen regarding which should be considered uremic toxins [65].

Under physiological conditions, cells from the endothelial, epithelial and immune system are found to interact within the kidney. When a harmful stimulus, such as bacteria or their associated products, nephrotoxic drugs or other stimuli, are present at the endothelium, Toll-like receptors (TLR), NOD-like receptors (NLR) and the inflammasome of the immune system cells activate. This stimulates the release of proinflammatory cytokines, recruiting monocytes and neutrophils to migrate to kidney tissue [66]. Besides, resident immune cells (mainly dendritic cells) activate and induce T-cell proliferation, in turn exacerbating cytokine release and inflammatory cascade. This exacerbated immune response may cause irreversible tissue damage and lead to loss of function in the affected organs [67].

Miyazaki et al. suggest that the organ dysfunction road may be associated with specific gene expression such as transforming growth factor B1 (TGF-β1), tissue metalloproteinase inhibitor-1 (TIMP-1) and pro-collagen α1 [68]. Intriguingly, the expression of these genes is suppressed by treatment with AST-120 [69].

Not only are MCP1 and its receptor, CCR2, the most studied cytokines. However, IL-8 and its receptor CXCL-8, IL-1, TNF-α, IL-1β, IL-6, TGF-β1 have also all been shown to cause mesangial and endothelial damage, inducing the expression of cell adhesion molecules (ICAM-1) in mesangial cells, tubular epithelial cells and podocytes, causing infiltration by neutrophils and monocytes [70] or inducing ROS synthesis [71]. IL-1β was found to correlate positively with IS and E-selectin in sera from 32 patients with AKI after cardiac surgery [72], a finding consistent with animal models of IRI. In vitro, these effects are attenuated by inhibiting the signaling pathway of MAPK.

Il-120, in particular, has been linked to tubular cell death, tubulointerstitial fibrosis and inflammation during the progression of AKI to CKD [73]. Additionally, IL-17 is implicated in the accelerated decline of acute liver failure in patients with AKI. IL-17 appears to be derived from the gut, since IL-17 levels are higher in portal vein and small intestine compared to systemic and liver levels. It has been suggested that the regulation of inflammatory response and cytokine release in the gut after AKI may have offer a therapeutical target to reduce complications following AKI [74].

Resistin, an adipose-tissue macrophage-derived cytokine, increases in patients with type 2 diabetes, obesity, CKD and sepsis. In vitro, it has been shown to inhibit neutrophil activation and chemotaxis [75]. In a study with plasma from 13 patients with AKI and septic shock, after 4 days under RRT, persistently elevated resistin was associated with neutrophil impairment by inhibiting actin polymerization [76]. Further research focusing on the effects of RRT in specific uremic toxins, other than cytokines, is needed.

### 5.2. Clinical Applications

Events of AKI have been associated with increased risk for adverse outcomes, including coronary events, ischemic or hemorrhagic stroke, dementia and even malignancy, supporting the idea that AKI impacts on prognosis beyond mortality [77].

Recently, tandem mass spectrometry has been successfully employed to measure a set of seven uremic toxins in patients with AKI after cardiac surgery [78]. Interestingly, this study found that uremic toxin concentrations were lower in AKI than in CKD, and the main toxins were indoxyl-sulfate and p-cresol. The rise in these toxins occurred parallel to the rise in creatinine. Commercially available methods are not yet available for widespread use, and further studies should include patients with other causes of AKI.

Several neurological derangements occur in AKI, however, the pathogenesis of uremic encephalopathy is not clear. In a rat model of ischemic AKI, an increase in neuronal picnosis and microgliosis was found, as well as increased levels of keratinocyte-derived chemokines and G-CSF in brain cortex and hippocampus, which may have a role in the blood–brain barrier [79]. Inflammation plays a role in the development of these lesions. It should be emphasized, these changes were absent in rats with isolated liver injury, which suggest that different effects originate according to the injured organ [80]. In rats undergoing bilateral nephrectomy, the main solutes that accumulate in CSF included IS, hippurate, TMAO and myo-inositol [81]. Studies in humans with AKI are scarce, however, in a population-based cohort study, AKI was found to increase the risk for dementia with a hazard ratio of 1.88 (95% CI 1.76–2.01) after adjustment for sex, age and comorbidities [82].

Cardiac effects may be mediated by IS and p-cresyl, which enter cardiac cells via OAT1/3. IS blocks a K+ channel that can have an arrythmogenic effect by delaying cardiac repolarization and prolonging QT interval. A reduction in IS in rat models can ameliorate cardiac fibrosis [83]. IS has also been implicated in vascular toxicity in patients undergoing hemodialysis, due to a high protein binding capacity, and it may have a role in bone disease [84]. Further studies are required to evaluate a benefit in reducing such toxins. It appears that cardiovascular and kidney dysfunction are main determinants of patients prognosis in the critical setting, as suggested by clinical observations using SOFA (Sequential Organ Failure Assessment) [85].

The gut-kidney axis is mediated by metabolites derived from intestinal microbiota, which can affect the kidneys and other organs. Increased levels of trimethylamine-N-oxide, a product of choline metabolism, were found to correlate with cardiovascular risk and death in a study of 283 individuals [86]. Along with p-cresyl sulfate and IS, these gut-derived uremic toxins are up-regulated in AKI and may accelerate atherosclerosis. Reversing microbiota dysbiosis is an appealing target. In a rat model of cisplatin-induced AKI, *Lactobacillus salivarius* prevented AKI by reducing levels of IS and p-cresyl sulfate. In vitro studies with human intestinal epithelial cells showed a reduced permeability, increased tight junction protein expression and reduced oxidative stress [87]. Rifaximin was found to correlate with a lower rate of AKI and need for RRT in a retrospective study with patients with cirrhosis. Studies of gut–kidney axis in human AKI are lacking [88]. A role for gut-derived short-chain fatty acids on renoprotection has been proposed, with immunomodulatory properties and protection during IRI [89].

## 6. Materials and Methods

Database (PubMed, Scopus and EBSCO) were searched using any combination of the keywords “acute kidney injury”, “uremic toxin” and “organ failure”, full texts were reviewed by the authors and then deemed suitable if presenting information on any organ effect of one or more uremic toxins, in the context of acute kidney injury. Information on any toxin led to inclusion in this review, presented in a biochemical order. Publication dates ranged from 1969–2021. Selected full-text articles were available in English or Spanish (except ref. [31] which is written in French).

## 7. Conclusions

The clinical associations and pathogenesis of AKI are complex, it is frequently encountered in critical patients and may have several contributing factors, including reduced blood flow, nephrotoxic exposure and systemic inflammatory response. An initial insult, such as hypoxia, may trigger MOF in this setting. However, there is a growing body of evidence surrounding the organ-specific effects of certain uremic toxins, which may be involved in organ cross-talk and mediate damage and recovery. Fluid overload, acid–base and electrolyte disturbances are well-known mediators for organ damage; however, end-organ damage may also result from effects derived from uremic toxins. Clinicians must always address the ‘bigger picture’ surrounding events of AKI and MOF, and further studies bridging the gap between basic research and clinical outcomes are required to clarify the complex relationships in organ crosstalk. Addressing MOF induced by AKI is of paramount importance for improving outcomes in critically ill patients.

## Figures and Tables

**Table 1:** Uremic toxins classification by EuTox.

**Small Water-Soluble Compounds (<500 D) with No Known** **Protein Binding**		**Protein-Bound Compounds**	**Middle Molecules (>500 D)**	
ADMA1 1-Methyladenosine Guanidine Orotidine 1-Methylguanosine Guanidinoacetate Oxalate 1-Methylinosine Guanidinosuccinate Phenylacetylglutamine 8-OH-2′Deoxyguanosine Guanilin Phenylethylamine Hypoxanthine Pseudouridine α-keto-δ-Guanidinovaleriate Inosine SDMA α-N-Acetylarginine Malondialdehyde Sorbitol Arabinitol Mannitol Taurocyamine Argininic acid Methylguanidine Thiocyanate Benzylalcohol	Myoinositol Threitol β-Guanidinopropionate N2-Dimethylguanosine Trimethylamine Creatine N4-Acetylcytidine Thymine Creatinine N6-Methyladenosine Uracil Cytidine N6-Threonylcarbamoyladenosine Urea Dimethylglycine N-Methyl-pyridone-carboxamide Uric acid Dimethylguanosine Nitrosodimethylamine Uridine Erythritol Nitrosomethylamine Xanthine γ-Guanidinobutyrate Orotic acid Xanthosine	Indoxyl sulfate * p-Cresyl * Homocysteine * p-OH-hippurate 2-Methoxyresorcinol Indole-3-acetate Pentosidine 3-Deoxyglucosone Phenol CMPF Kinurenine Phenylacetic acid Fructoselysine Kinurenic acid Glyoxal Melatonin Putrescine Hippuric acid Methylglyoxal Quinolinic acid Nɛ-Carboxymethyllysine Spermidine Hydroquinone Spermine	β2-Microglobulin Adiponectin Dinucleoside polyphosphates Methionine-enkephalin Adrenomedullin DIP I Motiline Atrial natriuretic peptide δ-Sleep-inducing peptide Neuropeptide Y Endothelin Octopamine β-Endorphin Ghrelin Orexin A β-Lipotropin Hepcidin Parathyroid hormone Basic fibroblast growth factor Hyaluronic acid Retinol binding protein Calcitonin-gene related peptide	Interleukin-1β Interleukin-6 Tumor necrosis factor α Substance P Cholecystokinin Clara cell protein Interleukin-18 Up4A Complement factor D Resistin κ-Ig Light chain Uroguanylin Cystatin C λ-Ig Light chain Vasoactive intestinal peptide Desacylghrelin Leptin

* Identified as relevant toxins found in experimental AKI (including cisplatin-induced AKI, nephrectomy, ischemia-reperfusion). Abbreviations: ADMA-1 (asymmetric dimethyl arginine), SDMA (symmetric dimethylarginine), CMPF (3-Carboxy-4-methyl-5-propyl-2-furanpropionate), DIP 1 (degranulation-inhibiting protein 1), Up4A (Uridine adenosine tetraphosphate).
